# Antibodies to Hepatitis B core antigen prevalence study in Kazakhstan

**DOI:** 10.1002/iid3.793

**Published:** 2023-03-27

**Authors:** Tatyana Savchuk, Yelena Grinvald, Mohamed Ali, Ramune Sepetiene, Saniya Saussakova, Kuralay Zhangazieva, Dulat Imashpayev, Saniya Abdrakhmanova

**Affiliations:** ^1^ Scientific‐Production Center of Transfusiology Nur‐Sultan Kazakhstan; ^2^ Abbott Laboratories GmbH Dubai UAE; ^3^ Abbott GmbH Max‐Planck‐Ring 2 Wiesbaden Germany; ^4^ Department of Public Health Astana Medical University Nur‐Sultan Kazakhstan

**Keywords:** blood donors’ virology markers, occult Hepatitis B, positive anti‐HBcor, prevalence of anti‐HBcor

## Abstract

**Introduction:**

Kazakhstan is being considered medium‐endemic for Hepatitis B virus infection (HBV). HBV remains transmissible by direct exposure to infected blood or organic fluids. This cross‐sectional study aimed to evaluate the prevalence of anti‐HBcore and the risk factors impacting positive anti‐HBcore markers among donors at Scientific‐Production Center of Transfusiology, Ministry of Healthcare of the Republic of Kazakhstan.

**Materials and Methods:**

The samples taken from blood donors were tested for anti‐HBcore, by the chemiluminescence immunoassay method on the Architect i2000SR (Abbott). In case of positive anti‐HBcore, the blood samples were further tested for anti‐HBs on the Architect i2000SR (Abbott). Alanine aminotransferase (ALT) indicators were tested by kinetic method on the Biosystems A25 analyzer. Statistical analysis was conducted using R software (version 4.1.1, 2021).

**Results:**

Five thousand seven hundred and nine people aged 18–66 years included in the study, the proportion of men and women was 68.17% and 31.83%, respectively. The average age of the participants was 35.7 ± 10.57 years. The prevalence of anti‐HBcore among donors was 17.2% (983). Among participants with elevated ALT (170), this marker was determined in 23%, and for donors with normal levels of ALT (5539)−17%. Participants with positive anti‐HBcore scores were on average older (41.8 vs. 34.4 years, *p* < .001) and Kazakhs (88.7% vs. 83.0%, *p* < .001) by nationality than study participants with negative results of anti‐HBcore.

**Conclusions:**

Anti‐HBcore prevalence in Kazakhstan (17.2%) compared with other countries (Croatia 7%, France 7%, Germany 9%, Iran 16%, Malaysia 20%, respectively) remains above average. Given the prevalence of HBV and risk factors, it is recommended to include an additional anti‐HBcore marker in the mandatory screening of donated blood in the Kazakhstan Republic and improve preventive measures to prevent HBV transmission by blood transfusions.

## Graphical abstract

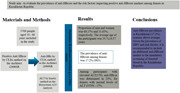

## 1 INTRODUCTION

Hepatitis B (HBV) is a contagious liver disease that results from infection with the Hepatitis B virus. The World Health Organization (WHO) estimated that approximately two billion people are carriers of HBV worldwide.^1^ Results of epidemiological studies have shown that about 240 million patients have chronic Hepatitis B infection (CHB).^2^, ^3^

HBV remains the infection transmissible by direct exposure to infected blood or organic fluids. The existing risk of transmission of HBV is associated with transfusion of blood taken from infected individuals during the window phase and presence of occult HBV.^4^, ^5^, ^6^, ^7^ High risk of transfusion‐transmitted infections stimulates the realization of the measures for blood safety increase.^8^

Routine HBV screening prefers testing HBsAg and anti‐HBs only, refusing to investigate other possible assays to detect occult Hepatitis B infection. Anti‐HBc is produced in infected individuals, both in chronic carrier state and at the end of acute resolving infection.^9^ Also, it is considered an effective marker for occult HBV infection and is an integral part of blood donors’ screening in many countries.

Historically, alanine aminotransferase (ALT) was used as a surrogate marker for viral hepatitis. Anti‐HBcore and ALT tests have been used in Brazil since 1993^10^ and Hungary since 2006.^11^ Results of a 10‐year surveillance program in Germany^12^ showed that anti‐HBcore screening introduced in 2006 has improved blood safety in the country. Study in Netherlands^13^ showed similar results. Since 2011 anti‐HBcore testing has enhanced the safety of the Dutch blood supply but still remains difficult to determine anti‐HBcor reactivity and occult Hepatitis B infection (OBI) due to the variable complexity of the immune response.

The study by Mosley et al.^14^ demonstrated that anti‐HBcore screening would reduce the amount of HBV to be processed by virus inactivation and increase the content of anti‐HBs in plasma pools in the United States.

Similarly, Souan et al.^15^ showed the importance of screening for anti‐HBcore to improve blood and platelets safety in Jordan. Study carried out by Rodella et al.^16^ reviewed the importance of the quantitative determination of anti‐HBcore in provision of additional information and may be useful in the differential diagnosis of acute and chronic HBV infections and in the follow‐up of chronically infected patients.

Kazakhstan falls into the category of countries with medium endemicity (2%–7%) for HBV infection.^17^ In 2020 about 23,000 HBV cases were registered, the peak of incidence was defined in West Kazakhstan, Kyzylorda region, and Nur‐Sultan city.^18^ Approximately 21,000 patients have CHB. In contrast, anti‐HBcore screening for blood donors is not confirmed in Kazakhstan.

The objective of this study was to determine the prevalence of anti‐HBcore among a subpopulation of blood donors and to evaluate the predominant risk factors associated with positive anti‐HBcore markers.

## 2 MATERIALS AND METHODS

### 2.1 Study design

A cross‐sectional study was conducted in 2021 in the Scientific‐Production Center of Transfusiology of the Ministry of Healthcare in Kazakhstan. Informed consent was obtained from each prospective blood donor recruited into the study. Ethics commission approved the study (Decision of EC #5 from August 20, 2020).

### 2.2 Inclusion and exclusion criteria

Participants who met the criteria donate blood, that is, no younger than 18 years old, no blood donation in the last 3 months, weigh over 50 kg, have not had Hepatitis B, C, HIV (AIDS), syphilis, parasitic diseases, and cancer. Donors with adequate hemoglobin (blood count) to donate (≥125 g/L for females and ≥135 g/L for males). All participants were healthy volunteers declared good health. Potential donors who did not meet the criteria for blood donation stated in the inclusion criteria were excluded from the study.

### 2.3 Laboratory measurements

**Figure 1:**
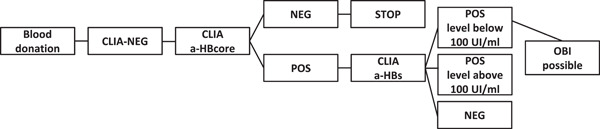
Algorithm for testing donors for the anti‐HBcore. OBI, occult Hepatitis B infection. POS, positive result; CLIA, chemiluminescence immunoassay; NEG, negative result.

The samples taken from blood donors were tested for anti‐HBcore, by chemiluminescence immunoassay method on the Architect i2000SR (Abbott). In case of positive anti‐HBcore, the blood was further tested for anti‐HBs on the same platform (Figure 1).

ALT was tested by kinetic method on the Biosystems A25 analyzer. The presence/absence of HIV, HBV DNA, and HCV were evaluated by polymerase chain reaction techniques in pools of six samples using the Cobas TaqScreen MPX Test v.2.0 multiplex test.

### 2.4 Statistical analysis

Statistical analysis was conducted using R software (version 4.1.1, 2021).^19^ Descriptive statistics were reported as proportions (%) for categorical variables and as mean ± standard deviation or median with interquartile ranges in parentheses for continuous variables. The normality of the distribution was tested using the Kolmogorov–Smirnov test. *χ* ^2^ or Fisher's exact tests for categorical variables and Student's *t* test or Mann–Whitney *U* test for continuous independent variables were conducted, as appropriate. Statistically significant results were considered values below *p* ≤ .05.

## 3 RESULTS

Five thousand seven hundred and nine participants were consecutively enrolled in this study. The sample was composed of 68.17% (3139) males and 31.83% (1466) females. The average age of the participants was 35.69 ± 10.57 years ranging from 18 to 66 years. The majority of the participants were of Kazakh origin (84%).

Analysis of anamnestic data showed that less than 2% of participants had a family member with hepatitis in the last 6 months, and more than 1% of respondents had been transfused with blood or its components in the last 12 months (1.2%).

**Figure 2:**
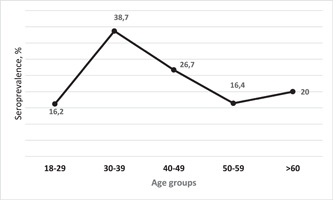
Age‐specific distribution of anti‐HBcore seroprevalence.

The prevalence of anti‐HBcore among study participants was 17.2% (983). The highest prevalence of anti‐HBcore was observed among the age group of 30–39 years old (Figure 2).

Participants with positive anti‐HBcore scores were on average older (41.8 vs. 34.4 years, *p* < .001), Kazakh origin (88.7% vs. 83.0%, *p* < .001) compared with participants with negative anti‐HBcore results, respectively (Table 1).

**Table 1:** Data for descriptive analysis.

Variables	Total (*N* = 5709)	anti‐HBcore		*p* Value
		Negative (*n* = 4726)	Positive (*n* = 983)	
Age, mean (SD))	35.69 (10.57)	34.42 (10.33)	41.75 (9.54)	<.001
Gender, *n* (%)				.46
Females	1466 (31.8)	1220 (32.1)	246 (30.7)	
Males	3139 (68.2)	2583 (67.9)	556 (69.3)	
Ethnicity, *n* (%)				<.001
Kazakh	3855 (84.0)	3146 (83.0)	709 (88.7)	
Russian	415 (9.0)	364 (9.6)	51 (6.4)	
Other ethnicities	320 (7.0)	281 (7.4)	39 (4.9)	
Have family members had hepatitis in the last 6 months? *n* (%)				.41
Yes	68 (1.6)	59 (1.7)	9 (1.2)	
Not	4096 (98.4)	3367 (98.3)	729 (98.8)	
Having transfusion of donated blood or its components in the last 12 months, *n* (%)				.57
Yes	56 (1.2)	44 (1.1)	12 (1.4)	
Not	4653 (98.8)	3835 (98.9)	818 (98.6)	
Having intravenous or intramuscular injections, acupuncture, tattoos, or piercings in the last 4 months				.71
Yes	247 (5.2)	201 (5.2)	46 (5.6)	
Not	4458 (94.8)	3678 (94.8)	780 (94.4)	
Having surgical interventions (including cosmetic surgery or organ removal), *n* (%)				.18
Yes	722 (15.3)	608 (15.7)	114 (13.7)	
Not	3989 (84.7)	3273 (84.3)	716 (86.3)	

*Note*: Missing answers: for gender—1104 (19.3%); for ethnicity—1119 (19.6%), for family history of hepatitis—1545 (27.1%), for blood transfusions—1000 (17.5%), for intravenous or intramuscular injections, acupuncture, tattoos or piercings—1004 (17.6%), for surgical interventions—998 (17.5%).

**Figure 3:**
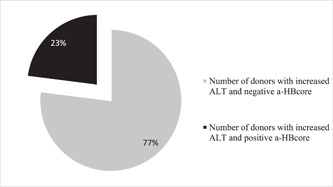
Indicators of anti‐HBcore detection among donors who had elevated ALT levels. ALT, alanine aminotransferase.

**Figure 4:**
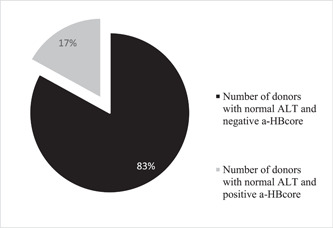
Indicators of anti‐HBcore detection among donors who had normal ALT indicators. ALT, alanine aminotransferase.

Among participants with elevated ALT (170), positive anti‐HBcore marker was determined in 23% (39) participants (Figure 3), and in 17% (944) of donors with normal levels of ALT (5539) (Figure 4).

**Figure 5:**
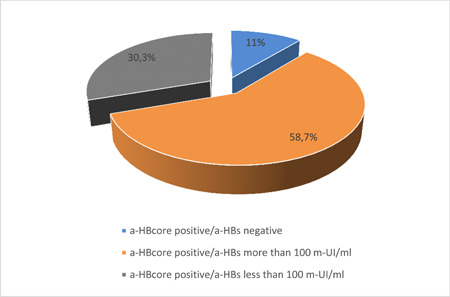
Prevalence of anti‐HBcore and anti‐HBs markers.

Our interpretation of the results was done according to the WHO recommendations^20^: a level of anti‐HBs equal to 100 mIU/mL is usually accepted as the lowest protective level in the context of blood screening, positive both anti‐HBcore and anti‐HBs less than 100 mIU/mL evaluated as indicators for blood rejection. Anti‐HBcore positive samples present antibodies against HBsAg found to have 89% (875) along with anti‐HBs positive markers (Figure 5). The number of donors with anti‐HBs titer less than 100 mIU/mL was 298.

## 4 DISCUSSION

We have investigated the seroprevalence of anti‐HBcore and anti‐HBs among healthy population—blood donors in Kazakhstan. The prevalence of anti‐HBcore among the study participants was found to be 17.2%. Results of testing of anti‐HBcore‐positive samples for the presence of antibodies to HBsAg indicated that 89% donors have positive anti‐HBs marker.

Houareau et al.^12^ estimated 0.22% of screened donations were anti‐HBcore reactive but negative for HBsAg. Study conducted by Villar et al.^21^ indicated overall prevalence of anti‐HBcore and anti‐HBs are 14.5%, and 40.9%, respectively. Higher anti‐HBcore prevalence was associated with older age (41 years and above). In our study, the seroprevalence of anti‐HBcore was low at the younger age group, then reached peak in the age group of 30–39 years old. We assume that it might be associated with the introduction of universal HBV vaccination program started in 1998.^22^

The prevalence of anti‐HBcor in Malaysia is 20%.^23^ The risk of anti‐HBcore seropositivity significantly increased in older donors and is 53% higher among males than females. However, according to the results of a study conducted in Almaty and published in 2007, total anti‐HBcore was similar for women and men within population of Russians and Kazakhs.^24^ 9.8% of Indian patients were tested to be positive for anti‐HBcore marker only.^25^

Ye et al.^26^ demonstrated the rate of anti‐HBcore positivity increased steadily with age, ranging from the age group younger than 30 years to the age group up to 50 years. Around 70% of patients carried anti‐HBs, which is similar to our findings. Younger donors with mean age of 25 years were exposed to HBV to a lesser extent compared with those with a mean age of 29 years in Pakistan blood donors’ study.^27^ Results of study carried out by Muselmani et al.^28^ suggest to have anti‐HBcore as additional screening test for blood donors in Syria to reduce the risk of HBV transmission. In Spain, the rate of anti‐HBcore positive and HBsAg negative cases is approximately 10% of adults in 26–65 years old group.^29^

Aguia et al.^30^ suggest approximately from 33% to 50% of Hepatitis B cases that could be transmitted by transfusion of blood with HBsAg negative donors could be prevented by anti‐HBcore screening.^30^ In Jordan, the prevalence of anti‐HBcore among blood donors was 6.04%.^12^

Zhou et al.^31^ demonstrated that positive anti‐HBcore results correlate directly with hepatic inflammation in CHB patients with normal ALT. In our study, a positive anti‐HBcore marker was determined in 23% of participants with elevated ALT, and in 17% of donors with normal levels of ALT. Before the NAT implementation, ALT was widely used in routine practice and called to be a surrogate hepatic infection marker. Despite various updated testing algorithms, ALT value for the detection of hepatitis or any other liver inflammation in its early stage is under debates, otherwise more positive opinions than negative outcomes for clinical proof have no doubts.^32^, ^33^

This study has several strengths. The large sample size (5709 participants) allowed for high statistical power to measure prevalence with greater precision. However, a limitation of the study must be considered. Risk factors impacted the study population were collected by donors’ self‐assessment and vaccination records were not assessed.

## 5 CONCLUSIONS

Anti‐HBcore prevalence in Kazakhstan health people population was found to be 17.2%. Comparing with other countries: Croatia 7%, France 7%, Germany 9%, Iran 16%, and Malaysia 20% our results remain above average. Given the prevalence of HBV, it is recommended to include an additional anti‐HBcore marker in the mandatory screening algorithm of donated blood in the country improving preventive measures for HBV transmission.

## AUTHOR CONTRIBUTIONS

**Tatyana Savchuk**: Data curation; methodology; supervision; writing—review and editing. **Yelena Grinvald**: Formal analysis; investigation; software; writing—review and editing. **Mohamed Ali**: Funding acquisition; writing—original draft. **Ramune Sepetiene**: Conceptualization; resources; writing—review and editing. **Saniya Saussakova**: Conceptualization; formal analysis; project administration; writing—review and editing. **Kuralay Zhangazieva**: Data curation; validation; visualization; writing—original draft. **Dulat Imashpayev**: Methodology; resources; visualization; writing—review and editing. **Saniya Abdrakhmanova**: Conceptualization; investigation; software; writing—review and editing.

## CONFLICT OF INTEREST STATEMENT

The authors declare no conflict of interest.

## Data Availability

Full data are available upon request.
